# Effects of High-Volume Versus High-Load Resistance Training on Skeletal Muscle Growth and Molecular Adaptations

**DOI:** 10.3389/fphys.2022.857555

**Published:** 2022-03-11

**Authors:** Christopher G. Vann, Casey L. Sexton, Shelby C. Osburn, Morgan A. Smith, Cody T. Haun, Melissa N. Rumbley, Petey W. Mumford, Nathan T. Montgomery, Bradley A. Ruple, James McKendry, Jonathan Mcleod, Adil Bashir, Ronald J. Beyers, Matthew S. Brook, Kenneth Smith, Philip J. Atherton, Darren T. Beck, James R. McDonald, Kaelin C. Young, Stuart M. Phillips, Michael D. Roberts

**Affiliations:** ^1^School of Kinesiology, Auburn University, Auburn, AL, United States; ^2^Duke Molecular Physiology Institute, Duke University School of Medicine, Duke University, Durham, NC, United States; ^3^Fitomics, LLC, Pelham, AL, United States; ^4^Department of Kinesiology, Lindenwood University, St. Charles, MO, United States; ^5^Department of Kinesiology, McMaster University, Hamilton, ON, Canada; ^6^MRI Research Center, Auburn University, Auburn, AL, United States; ^7^MRC-ARUK Centre of Excellence for Musculoskeletal Ageing Research, Clinical, Metabolic, and Molecular Physiology, University of Nottingham, Nottingham, United Kingdom; ^8^Edward Via College of Osteopathic Medicine – Auburn Campus, Auburn, AL, United States

**Keywords:** higher-load resistance training, higher-volume resistance training, muscle hypertrophy, non-myofibrillar protein, myofibrillar protein

## Abstract

We evaluated the effects of higher-load (HL) versus (lower-load) higher-volume (HV) resistance training on skeletal muscle hypertrophy, strength, and muscle-level molecular adaptations. Trained men (*n* = 15, age: 23 ± 3 years; training experience: 7 ± 3 years) performed unilateral lower-body training for 6 weeks (3× weekly), where single legs were randomly assigned to HV and HL paradigms. Vastus lateralis (VL) biopsies were obtained prior to study initiation (PRE) as well as 3 days (POST) and 10 days following the last training bout (POSTPR). Body composition and strength tests were performed at each testing session, and biochemical assays were performed on muscle tissue after study completion. Two-way within-subject repeated measures ANOVAs were performed on most dependent variables, and tracer data were compared using dependent samples t-tests. A significant interaction existed for VL muscle cross-sectional area (assessed *via* magnetic resonance imaging; interaction *p* = 0.046), where HV increased this metric from PRE to POST (+3.2%, *p* = 0.018) whereas HL training did not (−0.1%, *p* = 0.475). Additionally, HL increased leg extensor strength more so than HV training (interaction *p* = 0.032; HV < HL at POST and POSTPR, *p* < 0.025 for each). Six-week integrated non-myofibrillar protein synthesis (iNon-MyoPS) rates were also higher in the HV versus HL condition, while no difference between conditions existed for iMyoPS rates. No interactions existed for other strength, VL morphology variables, or the relative abundances of major muscle proteins. Compared to HL training, 6 weeks of HV training in previously trained men optimizes VL hypertrophy in lieu of enhanced iNon-MyoPS rates, and this warrants future research.

## Introduction

Skeletal muscle hypertrophy has been defined as an increase in the weight or cross-sectional area of muscle (Tesch and Larsson, 1982; Folland and Williams, 2007), with the increased volume of muscle coming from an enlargement muscle fibers (Morpurgo, 1897; Gollnick et al., 1972; Goldberg et al., 1975). It is generally recognized that resistance training results in skeletal muscle growth through proportional increases in myofibrillar and non-myofibrillar protein content (Helander, 1961; Goldspink, 1964; Gordon et al., 1967; Seiden, 1976). Myofibril proteins are defined herein as the proteins that make up the rigid structure of muscle (e.g., dystrophin, actinin, titin, and nebulin) as well as contractile proteins (e.g., actin and myosin isoforms). In contrast, non-myofibrillar proteins are enzymes involved with signal transduction, energy synthesis and breakdown (e.g., sarcoplasmic and mitochondrial enzymes), and other metabolic processes (Haun et al., 2019b).

Recently, there has been interest regarding whether higher-load (HL) versus higher-volume (HV) resistance training elicits differential training adaptations at the macroscopic, molecular, and functional levels. HL training involves lifting heavier weights per set with fewer repetitions (e.g., 5 sets of 5 repetitions @ 85% of a 1-repetition maximum for a given exercise). HV training involves lifting lighter weights per set with more repetitions (e.g., 5 sets of 10–12 repetitions @ 60–65% of a 1-repetition maximum for a given exercise). HV training bouts yield a higher total volume load (the product of weight × total number of repetitions) where the total weight lifted is generally higher relative to HL training bouts. Research has typically suggested that HL training elicits superior increases in strength and muscle fiber hypertrophy compared to lower-load HV training (Fry, 2004). However, Mitchell and colleagues reported that 10 weeks of HL or HV resistance training led to similar increases in muscle hypertrophy as assessed through MRI and fiber histology (Mitchell et al., 2012). Subsequent literature indicates that both HL and HV training can: (i) elicit similar changes in skeletal muscle hypertrophy (assessed through either ultrasound or MRI; Schoenfeld et al., 2015; Jenkins et al., 2016; Morton et al., 2016; Ikezoe et al., 2017; Jenkins et al., 2017) and (ii) elicit similar strength adaptations (Ikezoe et al., 2017; Dinyer et al., 2019), although equivocal evidence exists suggesting HL training elicits superior strength adaptations (Schoenfeld et al., 2015; Jenkins et al., 2016, 2017). Reasons for similar outcomes between HL and HV training could be due to total volume lifted being comparable between paradigms. However, few HV versus HL studies have sought to modulate training loads with the intent of accumulating more training volume during HV conditions.

Our laboratory recently reported that 6 weeks of extremely HV resistance training decreased the relative abundances of myosin heavy chain and actin protein content per milligram of dry tissue (Haun et al., 2019a). Our findings, as well as those of others who have reported moderate-to-higher volume resistance training elicits similar molecular adaptations (MacDougall et al., 1982; Roth et al., 1999; Meijer et al., 2015), led us to postulate that a disproportionate increase in the sarcoplasmic space relative to myofibril protein accretion (i.e., sarcoplasmic hypertrophy) may be a training adaptation to HV resistance training (Roberts et al., 2020a). More recently, our laboratory demonstrated that lower volume, higher-load resistance training (3–5 sets of 2–6 repetitions at 65–90% 1RM) resulted in a maintenance of type I muscle fiber cross-sectional area (fCSA) while increasing type II fCSA. Additionally, no changes in non-myofibrillar protein concentrations were observed despite a modest but significant decrease in actin protein concentrations (Vann et al., 2020). While preliminary, these two studies from our laboratory suggest that HV resistance training may facilitate a more robust expansion of non-contractile proteins in myofibers, whereas HL training may promote a proportional increase in myofibril protein accretion with muscle growth. However, no single study to date has examined whether HV versus HL training differentially alter the molecular milieu in skeletal muscle; in particular the relative abundances of prominent myofibrillar proteins as well as the long-term synthesis rates of myofibrillar versus non-myofibrillar proteins.

The purpose of this study was to elucidate whether 6 weeks of unilateral HV versus HL lower-body resistance training differentially affected metrics of skeletal muscle hypertrophy, strength, and/or molecular variables assessed from skeletal muscle biopsies sampled from the vastus lateralis (VL). We sought to ensure HV training achieved more training volume relative to HL training. We hypothesized no differences would exist between HV and HL training when examining changes in VL muscle area assessed *via* magnetic resonance imaging (MRI), or VL thickness assessed *via* ultrasound. Additionally, we hypothesized that HL training would elicit superior increases in various indices of strength. However, we posited HV training would result in increased non-myofibrillar protein concentrations and a concomitant decrease in the relative abundances of contractile proteins, whereas HL training would result in no changes in these markers. Additionally, we hypothesized that the integrated non-myofibrillar (iNon-MyoPS) rates would be greater in HV versus HL training, whereas integrated myofibrillar protein synthesis (iMyoPS) rates would be greater in HL versus HV training. Finally, we aimed to determine if HV versus HL training adaptations persisted 10 days following the cessation training given that our laboratory and others have demonstrated features sarcoplasmic hypertrophy occur during ~8–10 days following 6–12 weeks of resistance training (Kadi et al., 2004; Haun et al., 2019a). Thus, all measures (except for protein synthesis assessments) were obtained prior to training as well as 3 days and 10 days following training.

## Materials and Methods

### Ethical Approval and Pre-screening

Prior to study initiation, this protocol was reviewed and approved by the Auburn University Institutional Review Board and was conducted in accordance to the standards set by the latest revision of the Declaration of Helsinki (IRB approval #: 19-245 MR 1907), except this study was not registered in a database.

College-aged resistance-trained men from the local community were solicited to participate in this study. Participants were provided informed consent documents, which clearly outlined all procedures of the study including the collection of muscle biopsies. In addition, participants were instructed that they were free to withdraw from the study at any time without jeopardy. Eligible participants that provided verbal and written informed consent and were screened 4–7 days prior to the start of the study. Participants had to be free of cardio-metabolic diseases (e.g., morbid obesity, type II diabetes, and severe hypertension), or any conditions that preclude the collection of a skeletal muscle biopsy. Participants were queried for the use of medications or performance-enhancing drugs, and none of the participants reporting using drugs for medical or recreational purposes. Additionally, training status for participants was determined by two criteria: (i) self-reported resistance training >1 year at least three times weekly, and (ii) a tested barbell back squat of ≥1.5× bodyweight [estimated from a 3 repetition maximum (3RM) test] in accordance to standards designated by the National Strength and Conditioning Association (NSCA; Haff et al., 2016). At the conclusion of the screening visit, participants were asked to maintain their current nutritional practices and to cease all training outside of the study.

### Study Design

**Figure 1:**
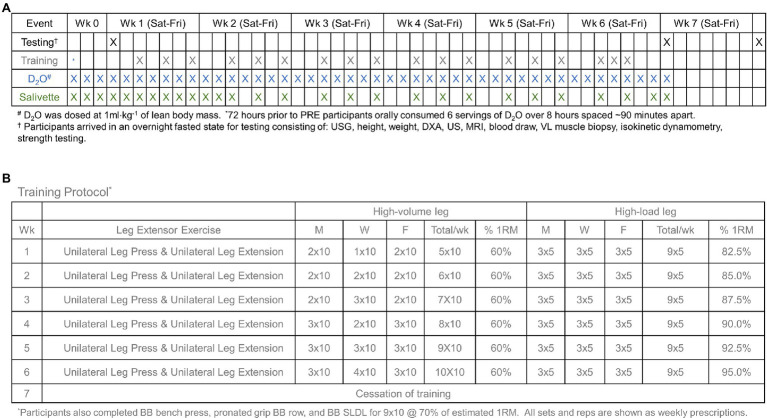
Study design. Panel **(A)** provides an overview of testing, training, D_2_O administration, and saliva collection times. Panel **(B)** provides a schematic of training by day and total training for each week. wk., week; D_2_O, deuterium oxide.

A schematic of the study design is provided in Figure 1. Briefly, participants performed a testing battery prior to the start of training (PRE), 72 h following the last bout of training after 6 weeks of unilateral lower-body resistance training (POST), and 10 days following the last bout of training (POSTPR). The testing batteries are detailed below, following a description of the training intervention and tracer methodologies.

### Resistance Training

Participants performed progressive unilateral lower-body resistance training (i.e., single-leg leg press and single-leg leg extension) 3 days per week in conjunction with compound upper body exercises (i.e., barbell bench press, pronated grip barbell row, barbell stiff-leg deadlift). Notably, participants were randomly assigned to lower-body training conditions prior to the start of the study, where some participants performed HV training on the left leg and HL training on the right leg or vice versa. All upper body exercises were performed for 3 sets of 10 repetitions at 70% of tested 1RM. Progression for the lower-body training can be found in Figure 1.

The HV training scheme was programed *a priori* considering that an individual engaged in a higher-volume training block would perform sets of 10 repetitions ~60% 1RM, and incrementally increase volume on a weekly basis. The HL training scheme was programed *a priori* considering that an individual engaged in strength training block would perform sets of 5 repetitions where the initial loads were ~ 80% 1RM, and incrementally increase training intensity on a weekly basis.

Auburn University staff supervised training, and weight lifted for each participant was logged in real time. Throughout training, we elected a systematic approach to adjust load within each training session if target repetitions per set were not met (e.g., a 5–10% reduction in load for the next set if 9/10 repetitions completed). However, this was only necessary on a few occasions, and most of the training was executed according to the planned study design.

### Tracer Protocol

Deuterium oxide (D_2_O; Cambridge Isotope Laboratories, Inc.; Andover, MA, United States) was provided to the participants 3 days prior to and over the first 6 weeks of the study at 1 ml•kg^−1^ of lean body mass. For rapid enrichment of deuterium (^2^H) participants were instructed to orally consume 6 doses of D_2_O over an eight-hour period, 3 days prior to the first data collection (PRE), and were instructed to consume a top-up dose daily thereafter consisting of one dose of D_2_O until data collection was performed at the conclusion of week 6 of the study (POST). Saliva samples were taken utilizing sterile salivettes (SARSTEDT AG &Co, Nümbrecht, Germany). Briefly, participants were instructed to chew on the cotton swab for 1 min and place the swab back into the top compartment of the salivette. This process was completed daily for the first 10 days of the study and every Monday, Wednesday, and Friday thereafter. Participants were instructed to place salivettes in their home freezers on days when saliva was donated outside of the laboratory. Samples were stored at −20°C until further processing as described below.

### Testing Sessions

#### Urine-Specific Gravity Testing for Adequate Hydration

Upon arrival to each testing session, participants submitted a urine sample (~5 ml) for urine-specific gravity (USG) assessment. Measurements were performed using a handheld refractometer (ATAGO; Bellevue, WA, United States). USG levels in all participants were ≤1.020 indicative of a euhydrated state and, thus, were considered adequately hydrated for further testing.

#### Body Composition Testing

Following hydration testing, participants underwent height and body mass assessments utilizing a digital scale (Seca 769; Hanover, MD, United States) with body mass collected to the nearest 0.1 kg and height to the nearest 0.5 cm. Participants were then subjected to a full body dual-energy X-ray absorptiometry (DXA) scan (Lunar Prodigy; GE Corporation, Fairfield CT, United States). Our laboratory (Kephart et al., 2016) has previously shown same day reliability of the DXA during test–calibrate–retest on 10 participants yields an intra-class correlation coefficient (ICC) of 0.998 for total body lean mass and an absolute standard error of the measurement (SEM) of 0.47 kg. Associated software was used to derive whole-body lean soft tissue mass (bone-free; abbreviated as LSTM) and fat mass. In addition, regions of interest were drawn around the upper left and right legs from the inguinal crease to the top of the knee to obtain upper leg LSTM, upper leg fat mass, and upper leg total mass (i.e., LSTM + fat mass + bone mass).

#### Measurements of Muscle Morphology

Following body composition testing, participants were tested for VL muscle thickness and muscle pennation angle *via* ultrasound. VL thickness of both legs was assessed by placing a 3–12 MHz multi-frequency linear phase array transducer (Logiq S7 R2 Expert; General Electric, Fairfield, CT, United States) midway between the iliac crest and lateral epicondyle of the femur. Measurements were taken from a standing position and participants were instructed to bear most of their weight on the leg contralateral to the leg being measured. VL pennation angles were taken immediately following thickness measures by placing the transducer longitudinally at the same site mentioned above. VL thickness was measured as the distance between the superficial and deep aponeurosis while VL pennation angle was measured as the angle of the deep aponeurosis as it relates to the individual fascicles. Estimated fiber length was calculated using methods similar to those described by Fukunaga et al. (1997) as seen in the equation below.


$$est.\mathrm{fiber}\;\mathrm{length}=\frac{a}{\cos\;\left(90{}^{\circ}-\theta \right)}$$

In the equation, a is equal to the distance between the superficial fascia and the deep aponeurosis and θ is equal to the angle of pennation. Importantly, to minimize variability in measurements as suggested in previous studies (Lohman et al., 2009; Lockwood et al., 2017), all measures were taken by the same investigator (S.C.O.), and this person in a test–retest validation on 10 participants had an ICC of 0.991 and an SEM of 0.06 cm. Critically, this investigator was not privy to the training condition for each participant’s leg. Moreover, the location of measurements was marked, using anatomical landmarks, by the investigator so that the subsequent MRI scans and muscle biopsies could be obtained from the same plane of measurement.

#### MRI for Muscle Cross-Sectional Area

Following ultrasound assessments, participants were shuttled to the Auburn University MRI Research Center to perform dual-leg mid-thigh MRI scans. All measurements were performed on a 3 T VARIO system (Siemens, Erlangen, Germany). Briefly, participants were placed in a supine position for 10 min to allow for body fluid stabilization to occur. A volume coil was used for RF transmit and body and spine array coils placed around the legs were used for signal reception. 3D gradient echo sequence (3D fast low angle shot) was used to acquire fat-suppressed images with the following parameters: TR/TE = 10/4.92 ms; flip angle = 10°; bandwidth = 510 Hz/pixel, in-plane resolution 1 mm×1 mm and slice thickness = 2.2 mm. An axial 3D 35.2 mm thick slab (a6 partitions) was placed to image both thighs with the thickness dimension carefully centered on the participant biopsy marking. Following the conclusion of the study, MRI scans were digitized using Osirix MD software (Pixmeo, Geneva, CHE), and software tools were used to manually trace the border of the VL yielding mCSA values. Measures were taken by the same investigator (R.J.B.) who did not possess knowledge of the training condition for each participant’s legs, and this person in a test–retest validation on 10 participants had an ICC of 0.999 and an SEM of 0.31 cm^2^.

#### Collection of Muscle Tissue

Following MRI scans, right and left leg VL muscle biopsies were collected using a 5-gauge needle under local anesthesia as previously described (Roberts et al., 2018, 2019). Immediately following tissue procurement, tissue was teased of blood and connective tissue, wrapped in pre-labeled foils, flash-frozen in liquid nitrogen, and subsequently stored at −80°C for processing described below.

#### Strength Testing

Following muscle skeletal muscle biopsies, participants underwent isokinetic dynamometry (Biodex System 4; Biodex Medical Systems, Inc., Shirley, NY, United States) for leg extensor peak torque and 3RM testing. For right and left leg extensor peak torque testing, participants were fastened to the isokinetic dynamometer. Each participant’s lateral epicondyle was aligned with the axis of the dynamometer, and seat height was adjusted to ensure the hip angle was approximately 90°. Prior to torque assessment, each participant performed a warm-up consisting of submaximal to maximal isokinetic knee extensions. Participants then completed five maximal voluntary isokinetic knee extension actions at 1.05 rad/s (60°/s) and 2.09 rad/s (120°/s). Participants were provided verbal encouragement during each contraction. The isokinetic contraction resulting in the greatest value was used for analyses. Peak torque measurements were not gravity-corrected. Following isokinetic dynamometry participants performed maximum strength testing for the exercises utilized over the duration of the study (single-leg leg press, single-leg leg extension, barbell bench press, pronated grip barbell row, and barbell stiff-leg deadlift). Briefly, participants performed 3 warm-up sets starting at ~50% of their self-selected opening weight for 10 repetitions, then 75% of their self-selected opening weight for 5 repetitions, and 90% of their self-selected opening weight for 3 repetitions. Following warm-ups, participants executed their opening attempt for 3 repetitions with 5–10% increases being made from there on until a 3RM was achieved. Given the advanced training status of participants, most had performed and were familiar with unilateral leg exercises, so this likely mitigated learning effects. All strength testing was performed by investigators holding the NSCA certified strength and conditioning specialist credential (C.G.V. and C.L.S.). Strength testing for single-leg leg press, single-leg leg extension, barbell bench press, pronated grip barbell row, and barbell stiff-leg deadlift occurred at PRE in order to properly program exercises throughout the study. However, given single-leg exercises were outcome variables of interest, these were the only two exercises that were strength tested at POST and POSTPR.

### Biochemical Assays

*Non-myofibrillar and myofibrillar protein isolation.* Isolation of protein fractions was performed using the proteomic validated “MIST” or “myofibrillar isolation and solubilization technique” (Roberts et al., 2020b). This method was validated through proteomic analysis showing that the myofibrillar fraction was exclusively enriched with myofibril proteins (i.e., MYH2, MHY1, MYH7, MYH4, ACTC1, and TTN), and none of these proteins were detected in the non-myofibril fraction. Additionally, several metabolic enzymes were enriched in the non-myofibrillar fraction (i.e., CKM, MB, ENO3, and PYGM), and these proteins were either not detectable or marginally present in the myofibrillar fraction. 1.7 ml polypropylene tubes were pre-filled with ice-cold buffer (300 μl; Buffer 1: 25 mm Tris, pH 7.2, 0.5% Triton X-100, protease inhibitors) and placed on ice. Skeletal muscle foils were removed from −80°C, placed on a liquid nitrogen-cooled ceramic mortar and pestle, and tissue was pulverized into 2–4 mm^3^ chunks. Chunks (~20 mg) were weighed using a scale with a sensitivity of 0.0001 g (Mettler-Toledo; Columbus, OH, United States) and placed into 1.7 ml polypropylene tubes with buffer and placed on ice. Samples were homogenized using tight-fitting pestles and centrifuged at 1,500 g for 10 min at 4°C. Supernatants (non-myofibrillar fraction) were collected and placed in new 1.7 ml polypropylene tubes on ice. As a wash step, the resultant myofibrillar pellet was resuspended in 300 μl of Buffer 1 and centrifuged at 1,500 g for 10 min at 4°C. The supernatant was discarded and the myofibrillar pellet was solubilized in 300 μl of ice-cold resuspension buffer (20 mm Tris–HCl, pH 7.2, 100 mm KCl, 20% glycerol, 1 mm DTT, 50 mm spermidine, protease inhibitors). After this step, tubes were visually inspected for insoluble connective tissue that may not have been teased out following tissue collection. Protein concentrations for the non-myofibrillar fraction were determined the same day as protein isolations to minimize freeze–thaw artifact. The myofibrillar fraction was prepared to analyze the relative abundances of major myofibril proteins and stored at −80°C until analysis occurred.

#### Determination of Non-myofibrillar Protein Concentrations

Non-myofibrillar protein resuspensions were batch-assayed for determination of protein concentration using a commercially available bicinchoninic acid (BCA) kit (Thermo Fisher Scientific; Waltham, MA, United States). Samples were assayed in duplicate using a microplate assay protocol where a small volume of sample was assayed (20 μl of 5× diluted sample + 200 μl Reagent A + B). The average duplicate coefficient of variation for non-myofibrillar protein concentration was 2.27%.

#### SDS-PAGE and Coomassie Staining for Relative Contractile Protein Abundance

Determination of the relative abundances of major myofibril proteins per mg wet tissue was performed as previously described by our laboratory and others (Cohen et al., 2009; Roberts et al., 2018; Dowling et al., 2019; Haun et al., 2019a). Briefly, SDS-PAGE sample preps were made using 10 μl resuspended myofibrils, 65 μl distilled water (diH2O), and 25 μl 4× Laemmli buffer. Samples (5 μl) were then loaded on precast gradients (4–15%) SDS-polyacrylamide gels in duplicate (Bio-Rad Laboratories) and subjected to electrophoresis at 180 V for 40 min using pre-made 1× SDS-PAGE running buffer. Following electrophoresis, gels were rinsed in diH2O for 15 min and immersed in Coomassie stain (LabSafe GEL Blue; G-Biosciences; St. Louis, MO, United States) for 2 h. Gels were then de-stained in diH_2_O for 60 min, and band densitometry was performed with a gel documentation system and associated software (ChemiDoc; Bio-Rad Laboratories, Hercules, CA, United States). Given that a standardized volume from all samples was loaded onto gels, band densities of different myofibril proteins were normalized to input muscle weights to derive arbitrary density units (ADU) per mg wet muscle. All values were then divided by the mean of the PRE time point to depict relative protein abundances of myosin heavy chain (MyHC). Our laboratory has reported that this method yields exceptional sensitivity in detecting 5–25% increases in relative actin and MyHC abundances (Roberts et al., 2018). Average duplicate coefficients of variation for relative protein abundances of actin, MyHC, tropomyosin, and troponin herein were 1.95, 1.90, 2.22, and 3.54%, respectively.

#### Six-Week Integrated Myofibrillar and Non-myofibrillar Protein Synthesis Rates

Protein isolations were performed using ~30 mg of tissue utilizing the MIST method as described above. Prior to preparation for tracer analysis, the non-myofibrillar protein fraction was lyophilized and precipitated in 1 ml of 1 M perchloric acid to form a pellet. The myofibrillar pellet was purified by adding 500 μl of DDH_2_O followed by vortexing for 5 s and centrifugation at 1,500 rpm at 4°C for 10 min. Following centrifugation, 1 ml of 0.3 M NaOH was added to the sample and then vortexed for 5 s followed by being placed in a heat block at 50°C for 30 min of which samples were vortexed for 5 s every 10 min. Samples then underwent centrifugation at 10,000 rpm at 4°C for 10 min. The supernatant (non-myofibrillar or myofibrillar fraction) was transferred into a 4 ml glass screw-top tube. 1 M perchloric acid was then added to tubes, and tubes were centrifuged at 2,500 rpm at 4°C for 10 min. The supernatant was removed, and the remaining pellet was washed in 70% ethanol and centrifuged at 2,500 rpm at 4°C for 10 min twice. Amino acids were extracted through the addition of 1 ml of 1 Dowex resin (50WX8-200 resin; Sigma-Aldrich) and 1 ml of 1 M HCl prior to heating at 110°C for 72 h. Cation exchange columns were used to isolate the free amino acids after which the amino acids were analyzed for deuterated-alanine content (^2^H-alanine) using a gas chromatography pyrolysis isotope ratio mass spectrometer. The amino acids were derivatized as their *n*-methoxycarbonyl methyl esters. Dried samples were suspended in 60 μl distilled water and 32 μl methanol, and following vortex, 10 μl of pyridine and 8 μl of methyl chloroformate were added. Samples were vortexed for 30 s and left to react at room temperature for 5 min. The newly formed *n*-methoxycarbonyl methyl esters of amino acids were then extracted into 100 μl of chloroform. A molecular sieve was added to each sample for ∼20 s before being transferred to a clean glass gas chromatography insert. Incorporation of deuterium into protein bound alanine was determined by gas chromatography–pyrolysis–isotope ratio mass spectrometry (Delta V Advantage) alongside a standard curve of known l-alanine-2,3,3,3-d4 enrichment to validate measurement accuracy of the instrument (Wilkinson et al., 2014).

Saliva deuterium analysis was performed to assess whole-body isotope enrichment. Briefly, the water phase of saliva was injected 6 times with the average of the last 3 injections being used for data analysis. The ^2^H isotope enrichments for both muscle and saliva were initially expressed as δ^2^H% and then converted to atom percent excess using standard equations as reported by Wilkinson et al. (2014). Fractional synthetic rates for the myofibrillar and non-myofibrillar protein fractions were calculated using the standard precursor-product method as described by other laboratories (Chinkes et al., 1993; Bell et al., 2019; McKendry et al., 2019).


$$FSR\;\left(\%day^{-1}\right)=\left[\frac{\left(E_{\mathrm{Ala}2}-E_{\mathrm{Ala}1}\right)}{E_{BW}\times t}\right]\times 3.7\times 100$$

In the equation above, E_Ala1_ and E_Ala2_ represent ^2^H enrichment at PRE and POST, respectively, (in atom percent excess) from skeletal muscle biopsies. E_BW_ is the average ^2^H enrichment (in atom percent excess) of total body water between time points and *t* is time in the number of days D_2_O was ingested. Multiplying by 3.7 adjusts for average ^2^H atoms that can be bound to alanine and multiplying by 100 converts this to a percentage per day (MacDonald et al., 2013; Wilkinson et al., 2014).

### Statistical Analyses

Statistical analyses were performed using SPSS (Version 26; IBM SPSS Statistics Software, Chicago, IL, United States), open-source software JASP (Version 0.11.0; JASP Team; 2019), and RStudio (version 1.1.463, R Foundation for Statistical Computing, Vienna, AT). Prior to analysis, assumptions testing for normality was performed using Shapiro–Wilk’s test for all dependent variables. If the assumption of heteroscedasticity was violated for repeated measures, a Greenhouse–Geisser correction factor was applied. Most dependent variables were analyzed using multi-factorial repeated measures ANOVAs, and if an interaction or main effect of time were observed (*p* < 0.05), manual Bonferroni adjustments were used to assess differences in dependent variables for leg or time. In this regard, significance for *post hoc* tests was established as *p* < 0.025 given that: (i) in the case of main time effects or interactions, two comparisons were made over time (POST versus PRE and POSTPR versus PRE), and (ii) in the case of interactions, two comparisons were made between legs at the POST and POSTPR time points. Tracer data were analyzed using dependent samples *t*-tests given that there was no time component to these data. Data are presented throughout as mean ± standard deviation (bar graphs) or box and whiskers plots including median (central horizontal line), 25th and 75th percentile (box), minimum and maximum values (vertical lines), and mean values (cross). Notably, a sample size of 15 participants was chosen *a priori* given the feasibility and logistics of performing individualized training sessions along with performing various techniques and assays that were resource intensive.

## Results

### Participant Characteristics

Baseline participant characteristics are presented in Table 1. Briefly, 15 college-age males (23 ± 3 years) with an average training age of 7 ± 3 years volunteered for this study. At PRE, participants weighed 89.5 ± 11.6 kg, with 69.1 ± 7.4 kg being LSTM and 17.3 ± 7.5 kg being fat mass, on average. Additionally, participants had an average relative squat to body mass ratio of 1.9× body mass (167 ± 34 kg).

**Table 1:** Participant characteristics.

Variable	Mean ± SD
Age (years)	23 ± 3
Height (cm)	182 ± 8
Weight (kg)	89.5 ± 11.6
Lean soft tissue mass (kg)	69.1 ± 7.4
Fat tissue mass (kg)	17.3 ± 7.5
Fat-free mass index	20.9 ± 2.2
Est. 1RM Squat (kg)	167 ± 34
Squat relative to body weight	1.9 ± 0.4

*N = 15 participants. Est. 1RM, estimated 1 repetition maximum. All measures taken prior to onset of training intervention*.

### Training Volume and Strength Metrics

**Figure 2:**
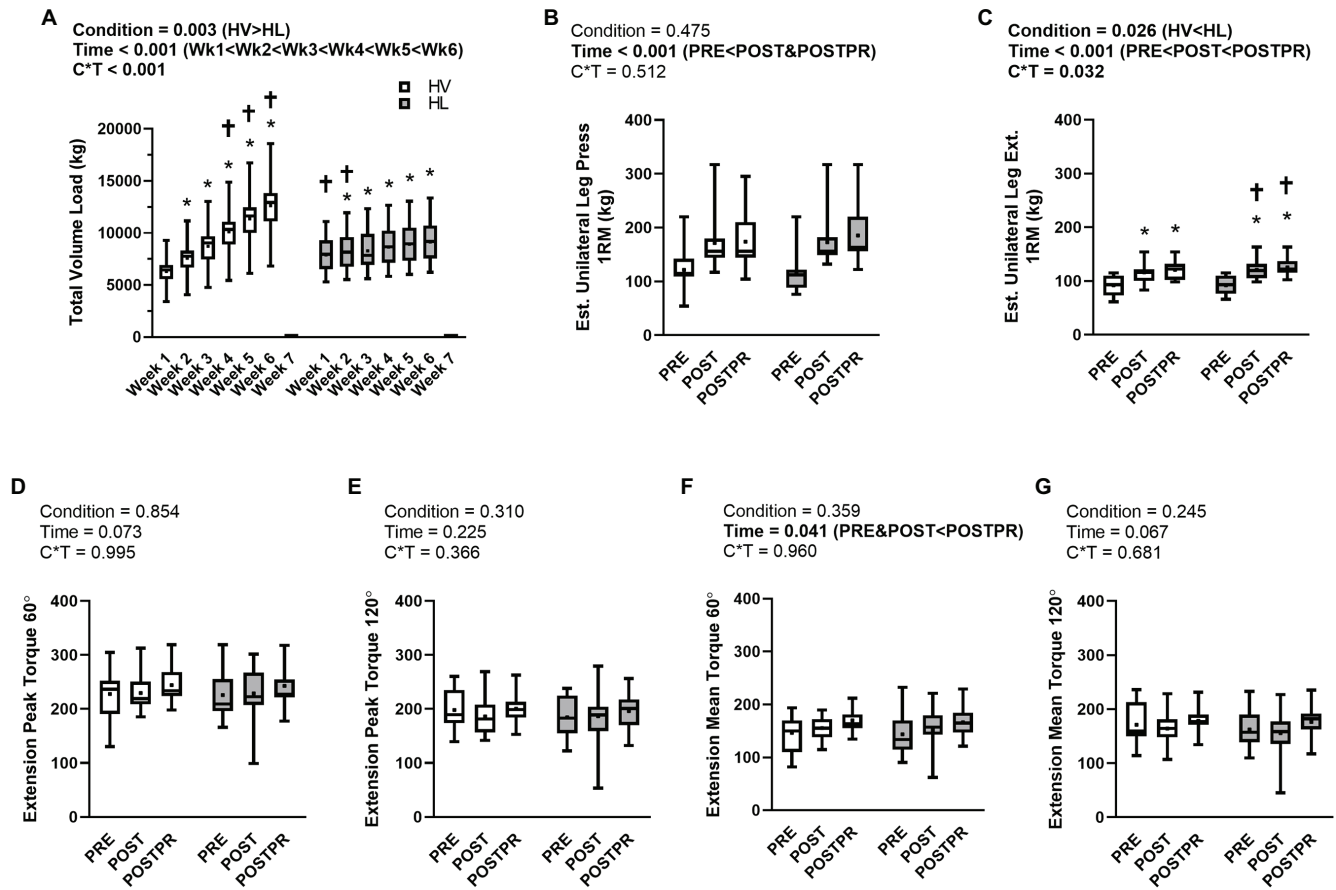
Training Volume and Strength Metrics. Legend: Data are presented as box and whiskers plots including median (central horizontal line), 25th and 75th percentile (box), minimum and maximum values (vertical lines), and mean values (cross) for training volume load (panel **A**), unilateral leg press (panel **B**), unilateral leg extension (panel **C**), knee extension peak torque at 60°/s (panel **D**), knee extension peak torque at 120°/s (panel **E**), knee extension mean torque at 60°/s (panel **F**), and knee extension mean torque at 120°/s (panel **G**). Abbreviations: HV, high-volume; HL, high-load. Symbols: ^*^indicates increase from PRE within condition; ^†^indicates HV > HL or HL > HV at a given time point.

Training volumes and strength metrics are presented in Figure 2. Data for 14 of 15 participants are presented for unilateral leg press and unilateral leg extension one repetition maximums (1RM) due to one participant feeling lower extremity discomfort at POST with these exercises.

There was a condition×time interaction observed for lower-body training volume (*p* < 0.001, $\eta_{p}^{2}$=0.914; Figure 2A). Additionally, there was a main effect of condition (*p* = 0.003, $\eta_{p}^{2}$=0.467) where the HV condition completed more volume than the HL condition (8,100 ± 480 kg versus 7,296 ± 421 kg, respectively). Lower-body training volume changed over time (*p* < 0.001, $\eta_{p}^{2}$=0.955, Figure 2A) and within each condition over time (HV: *p* < 0.001, $\eta_{p}^{2}$=0.952; HL: *p* < 0.001, $\eta_{p}^{2}$=0.954, Figure 2A). *Post hoc* analysis revealed lower training volumes at weeks 1 and 2 in the HV leg compared to the HL leg (*p* < 0.001 at each time point), no differences between conditions at week 3, and higher training volumes in the HV leg at weeks 4–6 as compared to the HL leg (*p* < 0.001 at each time point).

A condition×time interaction (*p* = 0.512, $\eta_{p}^{2}$=0.050, Figure 2B) was not observed for estimated unilateral leg press 1RM. Additionally, no main effect of condition (*p* = 0.475, $\eta_{p}^{2}$=0.040, Figure 2B) was observed. There was a main effect of time (*p* < 0.001, $\eta_{p}^{2}$=0.818, Figure 1B) where estimated unilateral leg press 1RM at POST (*p* < 0.001) and POSTPR (*p* < 0.001) were greater than PRE.

A condition×time interaction was observed for estimated unilateral leg extension 1RM (*p* = 0.032, $\eta_{p}^{2}$=0.265, Figure 2C). A main effect of condition (*p* = 0.026, $\eta_{p}^{2}$=0.328, Figure 2C) was also observed where the HL condition (grand mean = 113 ± 5 kg) estimated unilateral leg extension 1RM was higher than the HV condition (grand mean = 109 ± 5 kg). Estimated unilateral leg extension 1RM also changed over time (*p* < 0.001, $\eta_{p}^{2}$=0.885, Figure 2C) and within each condition over time (HV: *p* < 0.001, $\eta_{p}^{2}$=0.858; HL: *p* < 0.001, $\eta_{p}^{2}$=0.884). *Post hoc* analysis revealed no differences in estimated unilateral leg extension 1RM at PRE; however, the HL condition had higher values at POST and POSTPR compared to the HV condition (*p* < 0.025 at each time point). Given the significant interaction, we also calculated POST-PRE and POSTPR-PRE change scores for the HV and HL conditions and compared these scores using dependent samples t-tests as an additional *post hoc* analysis. Comparison of POST-PRE change scores indicated HL was greater than HV (30 ± 10 kg versus 25 ± 11 kg, respectively, *p* = 0.029). Similarly, comparison of POSTPR-PRE change scores indicated HL was greater than HV (34 ± 14 kg versus 29 ± 11 kg, respectively, *p* = 0.039). These results collectively indicate that HL training increased leg extensor strength more so than HV training.

There was no condition×time interaction observed for knee-extensor peak torque at 60°/sec (*p* = 0.995, $\eta_{p}^{2}$<0.001, Figure 1D) and 120° (*p* = 0.366, $\eta_{p}^{2}$=0.069, Figure 2E), or knee-extensor mean torque at 120°/sec (*p* = 0.681, $\eta_{p}^{2}$=0.027, Figure 2G). Additionally, there were no main effects of condition or time observed for the aforementioned variables. Knee-extensor mean torque at 60°/sec showed a significant time effect (*p* = 0.041, $\eta_{p}^{2}$=0.204, Figure 2F) where knee-extensor mean torque at 60°/sec trended higher at POSTPR than at PRE (*p* = 0.029) and POST (*p* = 0.043). However, these value of ps did not achieve a level of significance according to manual Bonferroni corrections (i.e., *p* < 0.025). There were no differences observed between PRE and POST (*p* = 0.805).

### Body Composition

PRE, POST, and POSTPR whole-body composition changes for all participants are presented in Table 2; notably, these data were derived from dual-energy X-ray absorptiometry (DXA) scans. Total body mass increased over time (*p* < 0.001, $\eta_{p}^{2}$=0.435), where POST (*p* = 0.001) and POSTPR (*p* = 0.012) body masses were greater than PRE. However, no differences were observed between POST and POSTPR body masses (*p* = 0.119). Whole-body LSTM increased over time (*p* = 0.003, $\eta_{p}^{2}$=0.338) where POST was greater than PRE (*p* = 0.002) and POSTPR (*p* = 0.014). No significant differences in LSTM were observed between PRE and POSTPR (*p* = 0.286). No differences were observed for DXA measured whole-body fat mass (*p* = 0.097).

**Table 2:** Body composition changes during training.

Variable	PRE	POST	POSTPR	ANOVA *p*-Value
	Mean ± SD	Mean ± SD	Mean ± SD	
Total Body Mass (kg)	89.3 ± 11.5	90.8 ± 11.9	90.4 ± 12.2	<0.001^†^
DXA Whole-body LSTM (kg)	69.1 ± 7.4	70.2 ± 7.5	69.5 ± 7.5	0.003^*^
DXA Whole-body Fat Mass (kg)	17.3 ± 7.5	17.4 ± 7.8	17.7 ± 7.8	0.097

N = 15 participants. DXA, dual-energy X-ray absorptiometry; LSTM, lean soft tissue mass. ^*^indicates measurement was higher at POST than at PRE and POSTPR (p < 0.05); ^†^indicates measurement was higher at POST and POSTPR than PRE (p < 0.05).

### Segmental Upper Leg Composition

**Figure 3:**
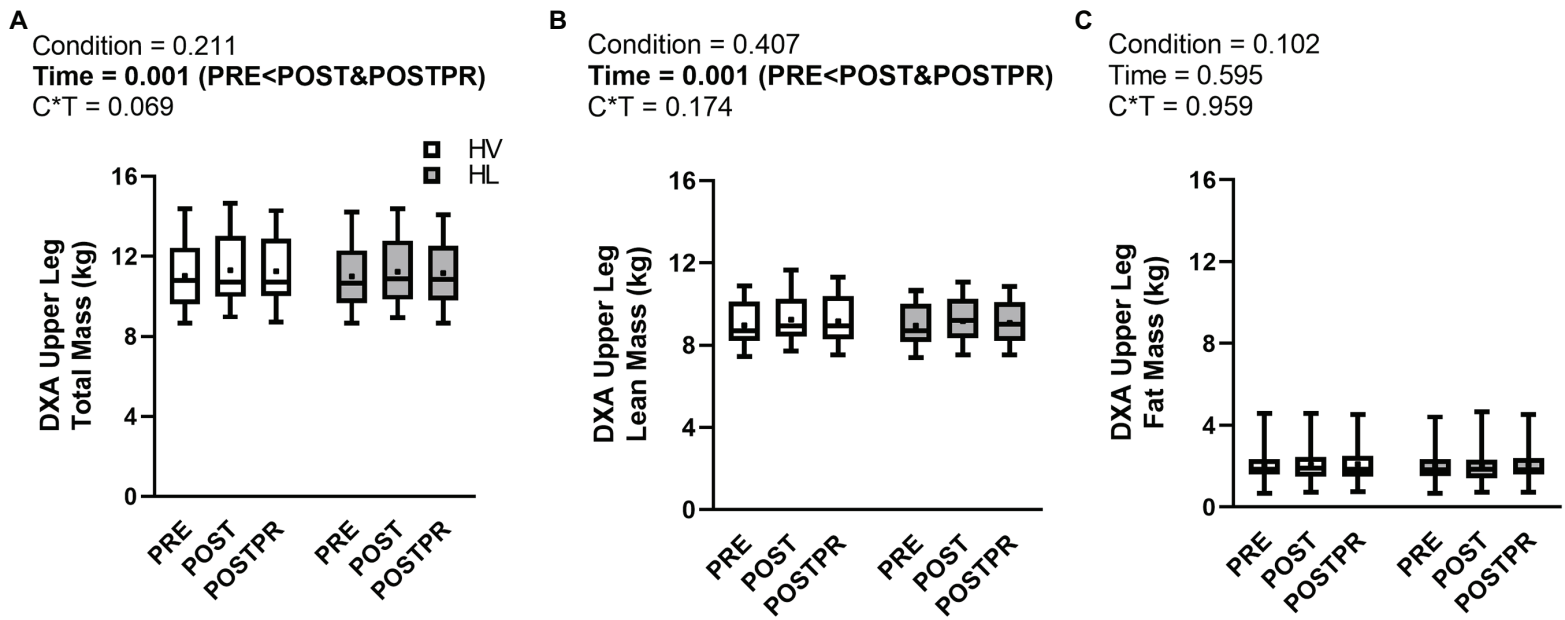
Segmental Upper Leg Composition. Legend: Data are presented as box and whiskers plots including median (central horizontal line), 25th and 75th percentile (box), minimum and maximum values (vertical lines), and mean values (cross) for DXA upper leg total mass (panel **A**), DXA upper lean mass (panel **B**), and DXA upper leg fat mass (panel **C**). Abbreviations: HV, high-volume; HL, high-load; DXA, dual-energy X-ray absorptiometry.

There were no condition×time interactions observed for DXA-derived upper leg mass (*p* = 0.069, $\eta_{p}^{2}$=0.173, Figure 3A), upper leg LSTM (*p* = 0.174, $\eta_{p}^{2}$=0.117, Figure 3B), or upper leg fat mass (*p* = 0.959, $\eta_{p}^{2}$=0.003, Figure 3C). A main effect of time was observed for upper leg mass (*p* = 0.001, $\eta_{p}^{2}$=0.392, Figure 3A) where POST (*p* = 0.002) and POSTPR (*p* = 0.013) were higher than PRE. No differences were observed between POST and POSTPR (*p* = 0.240). A main effect of time was observed for DXA upper leg LSTM (*p* = 0.001, $\eta_{p}^{2}$=0.418, Figure 3B) where POST (*p* < 0.001) and POSTPR (*p* = 0.002) were higher than PRE. No differences were observed between POST and POSTPR (*p* = 0.148). No main effects of condition (*p* = 0.102) or time (*p* = 0.595) were observed for DXA upper leg fat mass.

### Vastus Lateralis Muscle Morphology

**Figure 4:**
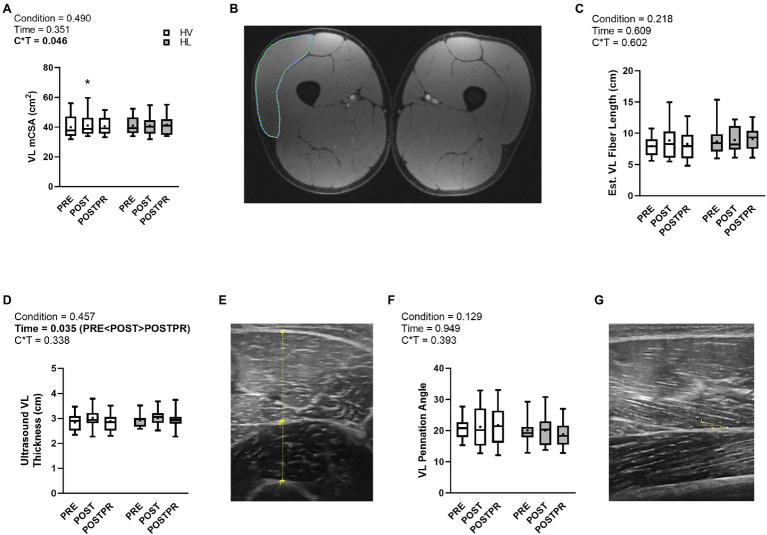
Vastus Lateralis Muscle Morphology. Data are presented as box and whiskers plots including median (central horizontal line), 25th and 75th percentile (box), minimum and maximum values (vertical lines), and mean values (cross) for VL mCSA (panel **A**), Est. VL fiber length (panel **C**), VL thickness (panel **D**), and VL muscle pennation angle (panel **F**). Representative images: Dual-leg MRI for VL mCSA (panel **B**), ultrasound cross section for VL thickness (panel **E**), ultrasound cross section for pennation angle (panel **G**). No significance was observed following decomposition of condition × time interaction for VL mCSA. Abbreviations: HV, high-volume; HL, high-load; VL, vastus lateralis; mCSA, muscle cross-sectional area; Est., estimated; Symbol: ^*^indicates increase from PRE within condition.

A condition×time interaction was observed for magnetic resonance image (MRI)-derived VL cross-sectional area (*p* = 0.046, $\eta_{p}^{2}$=0.211, Figure 4A); however, no main effects of condition (*p* = 0.490, $\eta_{p}^{2}$=0.037) or time (*p* = 0.351, $\eta_{p}^{2}$=0.077) were observed. *Post hoc* analysis revealed no differences between conditions at PRE (*p* = 0.246), POST (*p* = 0.673), or POSTPR (*p* = 0.247). However, POST was greater than PRE in the HV condition (*p* = 0.018), whereas this was not the case in the HL condition (POST versus PRE *p* = 0.475). Given the significant interaction, we also calculated POST-PRE and POSTPR-PRE change scores for the HV and HL conditions and compared these scores using dependent samples t-tests as an additional *post hoc* analysis. Comparison of POST-PRE change scores indicated HV was greater than HL (1.3 ± 2.1 cm^2^ versus 0.0 ± 2.1 cm^2^, respectively, *p* = 0.004). However, between-condition differences were not evident when comparing POSTPR-PRE change scores (HV = 0.7 ± 2.2 cm^2^, HL = 0.7 ± 1.8 cm^2^; *p* = 0.991). These results collectively indicate that VL hypertrophy occurred from PRE to POST in the HV versus HL condition.

There was no condition×time interaction (*p* = 0.338, $\eta_{p}^{2}$=0.075, Figure 4D) or main effect of condition (*p* = 0.457, $\eta_{p}^{2}$=0.040) observed for ultrasound measured VL thickness. VL thickness changed over time (*p* = 0.035, $\eta_{p}^{2}$=0.241) where POST values trended greater than PRE (*p* = 0.026) and were greater than POSTPR (*p* = 0.003). No differences were observed between PRE and POSTPR (*p* = 0.614). There were no interactions observed for muscle pennation angle of the VL (*p* = 0.393, $\eta_{p}^{2}$=0.064, Figure 4F) or estimated VL muscle fiber length (*p* = 0.602, $\eta_{p}^{2}$=0.036, Figure 4C). Additionally, there were no main effects of condition or time for the aforementioned variables (*p* > 0.05). Representative images from the MRI and ultrasound are provided in Figures 4B,E,G.

### Muscle Protein Adaptations

**Figure 5:**
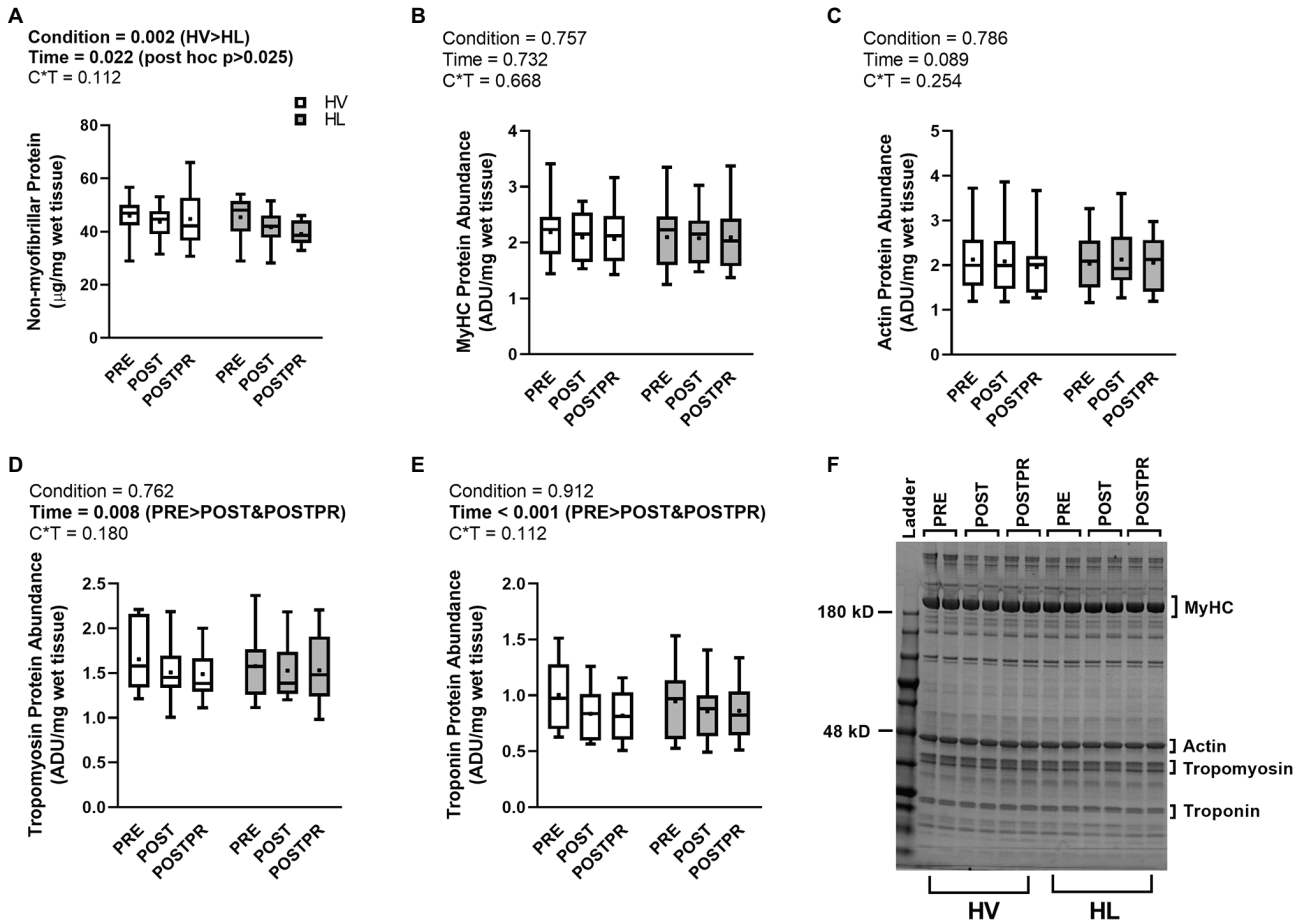
Muscle Protein Adaptations. Data are presented as box and whiskers plots including median (central horizontal line), 25th and 75th percentile (box), minimum and maximum values (vertical lines), and mean values (cross) for non-myofibrillar protein concentrations (panel **A**), MyHC protein abundance (panel **B**), actin protein abundance (panel **C**), tropomyosin protein abundance (panel **D**), and troponin protein abundance (panel **E**). Representative image: Coomassie blue stained poly-acrylamide gel for protein abundance (panel **F**). Abbreviations: HV, high-volume; HL, high-load; VL; MyHC, myosin heavy chain; ADU, arbitrary density units; kD, kilodalton.

There was no condition×time interaction observed for non-myofibrillar protein concentrations per mg of wet tissue weight (*p* = 0.112, $\eta_{p}^{2}$=0.159, Figure 5A). There was a main effect of condition (*p* = 0.002, $\eta_{p}^{2}$=0.497, Figure 5A) where non-myofibrillar protein concentrations in the HV group were higher than the HL group (44.8 ± 1.6 versus 42.6 ± 1.3 respectively). Additionally, there was a main effect of time (*p* = 0.022, $\eta_{p}^{2}$=0.239, Figure 5A) where PRE non-myofibrillar protein concentrations trended higher than POST (*p* = 0.038) and POSTPR (*p* = 0.032). However, these values of *p* did not achieve a level of significance according to manual Bonferroni corrections (i.e., *p* < 0.025). No differences in non-myofibrillar protein concentrations were observed between POST and POSTPR (*p* = 0.524).

There were no condition×time interactions observed for relative MyHC protein abundance per mg of wet tissue weight (*p* = 0.668, $\eta_{p}^{2}$=0.028, Figure 5B) or relative actin protein abundance per mg of wet tissue weight (*p* = 0.254, $\eta_{p}^{2}$=0.093, Figure 5C). Additionally, no main effects of condition or time were observed for these variables (*p* > 0.05). There was no condition×time interaction (*p* = 0.180, $\eta_{p}^{2}$=0.115, Figure 5D) or main effect of condition (*p* = 0.762, $\eta_{p}^{2}$=0.007, Figure 5D) observed for relative tropomyosin protein abundance per mg wet tissue weight. However, a main effect of time was observed for this variable (*p* = 0.008, $\eta_{p}^{2}$=0.294, Figure 5D) where PRE was greater than POST (*p* = 0.009) and POSTPR (*p* = 0.010). No differences were observed between POST and POSTPR (*p* = 0.704). There was no condition×time interaction (*p* = 0.112, $\eta_{p}^{2}$=0.145, Figure 5E) or a main effect of condition (*p* = 0.912, $\eta_{p}^{2}$=0.001, Figure 5E) observed for relative troponin protein abundance per mg wet tissue weight. A main effect of time was observed for this variable (*p* < 0.001, $\eta_{p}^{2}$=0.431, Figure 5E) where PRE was greater than POST (*p* < 0.001) and POSTPR (*p* = 0.005). No differences were observed between POST and POSTPR (*p* = 0.865). Figure 5F is a representative Coomassie gel.

### Six-Week Integrated Myofibrillar and Non-myofibrillar Protein Synthesis

**Figure 6:**
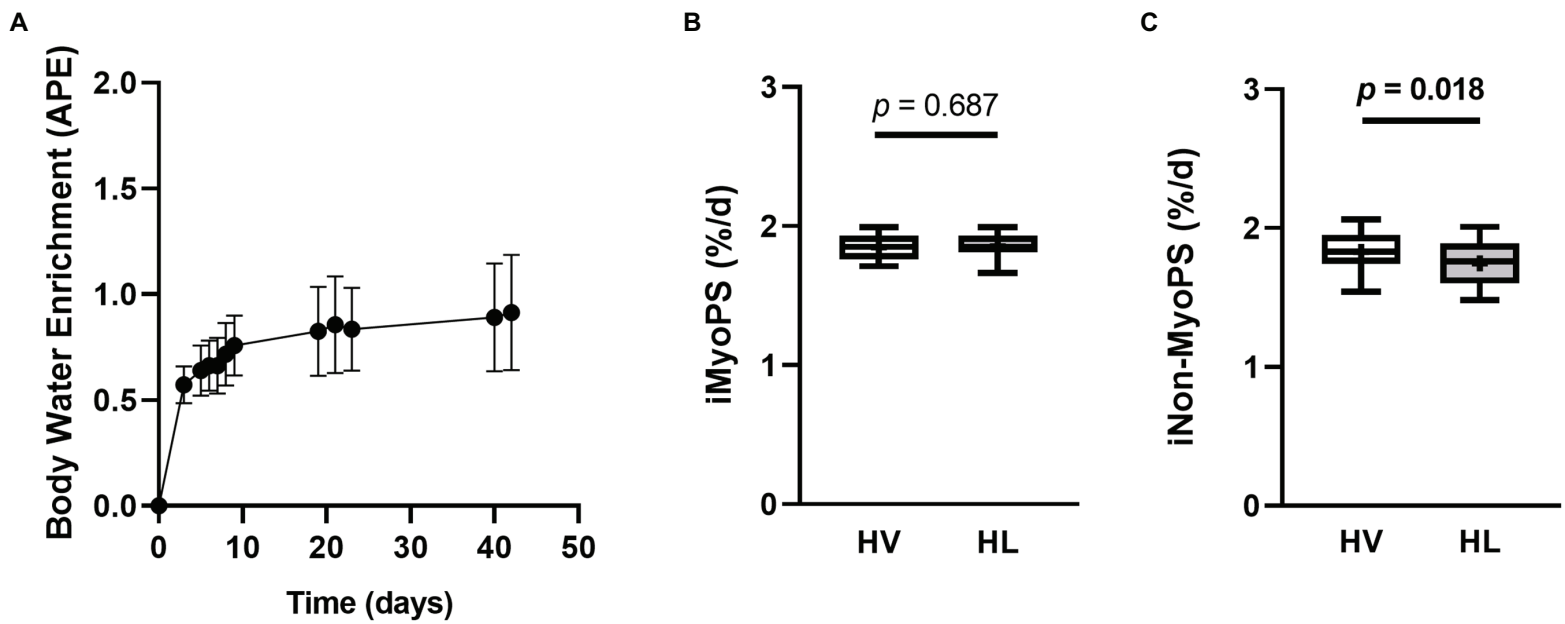
Six-week Integrated Myofibrillar and Non-Myofibrillar Protein Synthetic Rates. (Panel **A**) shows D_2_O enrichment from saliva analysis for 12 participants (means ± SD values). Data are presented as box and whiskers plots including median (central horizontal line), 25th and 75th percentile (box), minimum and maximum values (vertical lines), and mean values (cross) for iMyoPS (panel **B**) and iNon-MyoPS (panel **C**). No significant differences were observed for iMyoPS between conditions. iNon-MyoPS was significantly lower in the HL condition as compared to the HV condition. Abbreviations: HV, high-volume; HL, high-load; iMyoPS, integrated myofibrillar protein synthesis; iNon-MyoPS, integrated non-myofibrillar protein synthesis.

Figure 6A shows whole-body deuterium enrichment assessed *via* saliva samples for *n* = 12 participants. Following the loading phase (6 doses of D_2_O over an eight-hour period at 1 ml•kg^−1^ of lean body mass), deuterium enrichment increased significantly above baseline values (APE = 0.572 ± 0.087; *p* < 0.001). No difference was observed in iMyoPS rates between the HV and HL conditions (*p* = 0.687; *d* = −0.106; Figure 6B). A significant difference was observed for iNon-MyoPS rates where the HV condition exhibited a higher value than the HL condition (*p* = 0.018; *d* = 0.693; Figure 6C).

## Discussion

Chief findings from the current study include: (i) VL hypertrophy with HV training, but not HL training, from PRE to POST, (ii) greater increases in leg extensor strength with HL training, and (iii) iNon-MyoPS being greater in the HV versus HL condition. Notably, these results are for previously trained male participants, thus these findings may not hold true in other populations. The relevance of these as well as other findings are discussed below. A significant limitation is a lack of histology data detailing type I and II fiber type adaptations, and this is discussed later.

There is prior literature that has interrogated differences between HV and HL training paradigms. Holm and colleagues (Holm et al., 2008) reported that high-load (~70% 1RM) versus very low-load (~15.5% 1RM) leg extensor training increased quadriceps CSA; however, the change in the high-load condition was greater than the change in the low-load condition. Chestnut and Docherty (1999) reported similar increases in muscle CSA of the upper arm following 10 weeks of upper body resistance training using ~85% of 1RM for 6 sets of 4 repetitions versus ~70% for 3 sets of 10 repetitions. Mitchell and colleagues reported that performing three sets of knee-extensor training to fatigue at 30% or 80% of 1RM resulted in similar increases in quadriceps volume measured by MRI (Mitchell et al., 2012). Both modalities yielded greater quadriceps hypertrophy than performing one set at 80% 1RM to voluntary failure. Furthermore, a systematic review and meta-analysis conducted by Schoenfeld and colleagues concluded that similar skeletal muscle growth can be realized across a variety of loading ranges (Schoenfeld et al., 2017). While it is challenging to form a cohesive model based on our data and the literature cited above, our finding that VL mCSA increased from PRE to POST with HV training further implies that higher-volume training at loads corresponding to ~60% 1RM can be used to optimize hypertrophy in previously trained men. However, in accordance with the current data and some of these previously mentioned studies, the training loads utilized with HV training paradigms likely need to be between 30 and 85% 1RM to optimize hypertrophy.

As mentioned above, HL training increased leg extensor 1RM values more so relative to HV training. Several studies have examined changes in strength between different loading paradigms. Campos and colleagues reported high-load resistance training (3-5RM) over an 8-week period yielded greater leg extension strength increases compared to high-volume resistance training (20-28RM; Campos et al., 2002); however, no differences in strength adaptations were reported between the 3-5RM group and a third group which performed training using 9-11RM loads. Additionally, Jenkins et al. published two studies comparing 30% 1RM versus 80% 1RM leg extensor training (Jenkins et al., 2016, 2017). Results from both studies suggest that higher-load training elicited greater strength increases due to neural factors. Jessee et al. (2018) reported that unilateral training (4 sets to volitional failure) over an 8-week period resulted in greater strength adaptations for HL training (70% 1RM/no blood flow restriction) than low-load conditions with or without blood flow restriction. Furthermore, Schoenfeld and colleagues reported increased barbell back squat strength with lower (30–50% 1RM) and higher-load (70–80% 1RM) training, with higher-load training resulting in greater strength adaptations (Schoenfeld et al., 2015). When considering our findings in the context of these studies, it seems plausible that training at 60–90% 1RM over shorter-term periods may elicit similar strength alterations with certain strength tests; in this case the leg press and isokinetic dynamometer. However, given that leg extensor 1RM values increased more so with HL versus HV training and as implicated in prior research (Spitz et al., 2020), it also remains possible that strength adaptations for certain exercise tests that are practiced through higher-load training may also be optimized.

A novel aspect of the current study was to compare how HL versus HV training affected molecular markers from muscle biopsies. This interrogation was prompted by select literature suggesting that a disproportionate increase in non-contractile proteins in myofibers may occur following high-volume resistance training. However, as reviewed by Jorgenson and colleagues, several studies have shown that mechanical-load induced skeletal muscle hypertrophy is largely attributed to proportional increases in the contractile and non-contractile elements of the myofiber (Jorgenson et al., 2020). In the current study, no significant changes in the relative protein abundances of actin and MyHC were observed in either condition. Prior to discussing the implications of these data, it is important to understand the logistics of the contractile protein assay used herein, and readers are referred to a methods paper as well as a recent review on the topic from our laboratory for further details (Roberts et al., 2020a,b). In the presence of muscle hypertrophy (e.g., increases in VL mCSA), if sarcoplasmic protein concentrations and the relative abundances of contractile proteins remain unaltered from pre-to-post training then this likely indicates a proportional expansion of myofibril and non-myofibril components during growth; this being a phenomenon we have termed as “conventional hypertrophy” (Roberts et al., 2020a). On the other hand, if values, and in particular the relative abundances of myofibril proteins decrease from pre-to-post training then this indicates a “dilution” effect wherein hypertrophy occurs in the midst of sarcoplasmic (or fluid) expansion. Although our data largely imply conventional hypertrophy occurred with HV training, a handful of studies exist showing that a disproportionate increase in non-contractile proteins and cellular spacing may occur following months to years of resistance training (Penman, 1969; MacDougall et al., 1982; Toth et al., 2012; Meijer et al., 2015). Recently, our laboratory has reported decreases in the relative abundances of MyHC and actin protein abundances per mg of dry tissue weight following 6 weeks of extremely high-volume resistance training in previously trained college-aged men (Haun et al., 2019a). We previously posited that this was reflective of sarcoplasmic hypertrophy. Our laboratory subsequently reported that small (but significant) decrements occurred in actin protein abundance in previously trained college-aged males that partook in a 10-week low-volume, high-load training paradigm (Vann et al., 2020), and again we interpreted this as being reflective of sarcoplasmic hypertrophy. When considering the findings from both studies, we hypothesized that HV training in those with prior training experience might facilitate sarcoplasmic hypertrophy, whereas HL training may facilitate proportional accretion of contractile and non-myofibrillar proteins with whole-muscle hypertrophy (i.e., conventional hypertrophy). Although our current data disagree with prior findings from Haun et al., it is important to note the key differences that exist between the high-volume components of each study. In particular, Haun et al. used a 6-week intervention starting at 10 sets of 10 repetitions per week (for each exercise) and finishing with 32 sets of 10 repetitions per week where loads were standardized at 60% 1RM (Haun et al., 2018). The current study started the HV leg at 10 sets of 10 repetitions per week (split between two exercises) at week 1 and finished with 20 sets of 10 repetitions per week at week 6 where loads were standardized at 60% 1RM. Thus, although the HV leg was exposed to more training volume compared to the HL leg herein, the HV leg did not experience nearly the amount of volume as both legs incurred in the study by Haun et al. Moreover, the total training volume data in Figure 1 indicates that the HV leg was only exposed to ~11% more volume compared to the HL leg. We speculate that similar molecular adaptations between legs may have been due a relatively small difference in total training volume between legs throughout the duration of the study.

Despite the null findings discussed above, it is intriguing that HV training increased iNon-MyoPS rates versus HL training. This partially supports the notion that HV training may affect the non-myofibril protein pool more so than HL training. It is difficult to determine mechanisms associated with these observations given that time course biopsies were not procured to examine molecular signaling pathway differences between legs acutely following a single HV versus HL bout. We and others have shown that mTORC1 signaling markers, as well as the expression of mRNAs associated with skeletal muscle hypertrophy, are largely similar acutely following a HV versus LV training bout (Burd et al., 2010; Haun et al., 2017). Burd and colleagues also demonstrated that sarcoplasmic protein synthesis rates were elevated 24 h following a single HV versus HL exercise bout, and MAPK signaling was transiently elevated 4 h following the HV versus HL bout. While speculative, it may be possible that HV training herein stimulated MAPK signaling following each exercise bout more so than HL training, and this led to greater increases in iNon-MyoPS rates in the former condition. This hypothesis is supported by limited *in vitro* work demonstrating MAPK inhibition reduces protein synthesis rates (Servant et al., 1996; Kelleher et al., 2004). HV training may also increase intracellular calcium levels in a transient fashion more so than HL training, and heightened intracellular calcium levels have been shown to increase MAPK signaling (White and Sacks, 2010). These indirect lines of evidence lead to a hypothetical model where HV training, through elevated intracellular calcium concentrations and MAPK signaling, lead to greater increases in sarcoplasmic protein synthesis (i.e., iNon-MyoPS) rates relative to HL training. However, it has not been determined if elevated MAPK signaling in skeletal muscle leads to preferential increase in sarcoplasmic, versus myofibrillar, protein synthesis rates. Thus, this potential mechanism requires further investigation.

As with many studies examining the effects of training interventions, the present study is limited due to a small sample size. The procurement of skeletal muscle tissue *via* percutaneous muscle biopsy inherently has a finite tissue yield. We lacked an adequate amount of tissue to perform histology as we have done in the past with HV and HL training paradigms. Moreover, we recently developed a method to discriminate cell area occupied by myofibrils in type I and II fibers (Ruple et al., 2021), and this (along with tracking changes in type I and II fiber cross-sectional areas) would have added extraordinary insight to the current dataset. Data related to leg fluid shifts (e.g., leg segmental bioelectrical impedance spectroscopy or BIS) or tissue fluid content (e.g., lyophilization and comparing wet and dry tissue masses) were also not performed herein due to logistical constraints. Again, this remains an unresolved limitation. While protein synthesis rates were measured herein, it is notable that muscle protein breakdown rates were not assessed. The former are commonly measured, whereas the latter are rarely measured given the technical challenges that are often cited [reviewed in (Tipton et al., 2018)]. We speculate that protein breakdown rates are likely volume-dependent, and over longer time courses (i.e., > 6 weeks), this may affect phenotype outcomes given that net protein balance would be higher in HL versus HV training. However, no data exist supporting this contention, and this needs to be formally assessed. In spite of collecting training volume throughout the course of the study, we lack time under tension data and this would have been insightful to include in the current dataset. In spite of this limitation, Jenkins et al. (2017) have shown that individuals performing a similar type of unilateral HV training accumulated approximately three times the amount of time under tension relative to the HL-trained leg over a 6-week period. Thus, while we lack these data, we suspect that our participants experienced time under tension stimuli between legs. A final limitation of the current study is the length of training as well as our programming. With regard to the former, previous literature has shown 3–6 weeks of resistance training increases measures of hypertrophy in untrained to recreationally trained men (Seynnes et al., 2007; DeFreitas et al., 2011; Haun et al., 2018). In this regard, we posit that the training status of the cohort in the current study may have precluded our ability to detect any meaningful training adaptations over the 6-week training period. With regard to programming, we contend that a strength includes the real-world applicability; namely, HV and HL load progressions would likely follow similar patterns in recreational gym-goers. However, limitations to our approach include *a priori* programming being a bit arbitrary as well as weekly volume loads being more accelerated in the HV versus HL condition.

In conclusion, HV training elicited VL hypertrophy, whereas HL training resulted in a greater increase in leg extension strength. The current data challenge our prior muscle-molecular findings given that no alterations were observed in myosin heavy chain and actin protein abundances following either training protocol. However, the current iNon-MyoPS findings suggest some muscle-molecular differences exist between HV and HL training and warrant further research.

## Data Availability Statement

The raw data supporting the conclusions of this article will be made available by the authors, without undue reservation.

## Ethics Statement

The studies involving human participants were reviewed and approved by Auburn University Institutional Review Board. The patients/participants provided their written informed consent to participate in this study.

## Author Contributions

CV, SP, and MR devised the project aims and outcomes, and DB, JMcD, and KY provided critical insight. MR, SP, KS, and PA devoted significant resources to project outcomes. CV, CS, SO, MS, CH, MR, NM, BR, JMcl, and JMcK were involved with multiple aspects of data collection and analyses. AB, RB, and KY developed methods for MRI analyses. CV, SP, and MR primarily drafted the manuscript, and all authors edited the final manuscript for submission.

## Funding

Funding for assays and participant compensation was provided through discretionary laboratory funds from MR. Funding for MRI imaging was provided through discretionary laboratory funds from KY. Funding for deuterium oxide was provided through discretionary lab funds from SP. Additionally, a portion of CG Vann’s effort was funded through the National Institutes of Health (R01AG054840). The data that support the findings of this study are available from the corresponding author upon reasonable request.

## Conflict of Interest

CH was CEO of Fitomics, LLC while being involved with this study. However, Fitomics, LLC did not financially contribute toward study expenditures or any other aspect related to the study.

The remaining authors declare that the research was conducted in the absence of any commercial or financial relationships that could be construed as a potential conflict of interest.

## Publisher’s Note

All claims expressed in this article are solely those of the authors and do not necessarily represent those of their affiliated organizations, or those of the publisher, the editors and the reviewers. Any product that may be evaluated in this article, or claim that may be made by its manufacturer, is not guaranteed or endorsed by the publisher.
